# Spinnaker-sail sign in full-term neonates with spontaneous pneumomediastinum: a case study and scoping literature review

**DOI:** 10.1186/s12887-025-05641-5

**Published:** 2025-04-14

**Authors:** Ali Zamlout, Bushra Jamahiri, Elisar Jabbour

**Affiliations:** 1 Department of Pediatric Surgery, Tishreen University Hospital, Latakia, Syria; 2 Department of Pediatrics, Tishreen University Hospital, Latakia, Syria

**Keywords:** Neonate, Pneumomediastinum, Spontaneous pneumomediastinum, Spinnaker sail, Extrapleural air sign, Respiratory distress, Needle decompression, Chest tube

## Abstract

**Background:**

Pneumomediastinum is a condition that is occasionally observed in preterm neonates, characterized by the presence of free air within the mediastinal spaces. Spontaneous Pneumomediastinum (SPM) in full-term neonates is a rare form. The clinical spectrum ranges from asymptomatic cases to severe respiratory distress.

**Objective:**

To highlight the diagnostic challenges posed by the “spinnaker-sail sign”, and to create a stepwise framework for clinicians encountering similar cases.

**Methods:**

We present two cases of SPM in a full-term neonate. Case-1: a 2-day-old boy with respiratory distress exhibited on CXR a crescentic radiolucent configuration elevating the thymus from the pericardium (“spinnaker-sail” sign). CT demonstrated an extrapulmonary multiseptated cystic mass within the anterior mediastinum. The neonate was treated with supplemental oxygen and antibiotics, showing improvement by day 11. Case-2: a 6-hour-old boy presented with respiratory distress shortly after birth. CXR showed the spinnaker-sail sign, alongside a band of air overlying the left hemidiaphragm (“Extrapleural air” sign). Lateral projection revealed mediastinal air collection lifting the thymus from the pericardium and great vessels. He was managed with oxygen moisture and antibiotics, showing significant improvement by day 4.

**Discussion:**

The pathophysiology stems from uneven inflation and minute ruptures of immature alveoli, allowing air to leak through peribronchial and perivascular fasciae into the mediastinum. A fetal-remnant fascia entraps this air behind the thymus, constituting the “spinnaker-sail” appearance. The clinical course is typically benign. The management mainly involves supplemental oxygen and close monitoring. In severe cases, interventions such as needle decompression or chest tube insertion may be warranted.

**Conclusion:**

Unfamiliar radiographic patterns of PM in neonates pose diagnostic challenges and interventional hazards. Understanding the unique anatomy of the mediastinum in neonates is essential to formulate a proper diagnosis and management strategy.

**Clinical trial number:**

Not applicable.

## Background

Pneumomediastinum (PM, or mediastinal emphysema) is a condition characterized by free air within mediastinal spaces, occasionally observed in preterm neonates with respiratory pathologies or those on mechanical ventilation. Spontaneous PM (SPM) in full-term neonates is a rare form that, theoretically, results from non-traumatic ruptures of the bronchoalveolar walls, leading to air leakage into the mediastinum. Radiographically, it may appear as a unique crescent-shaped radiolucent entity, elevating the thymus from the pericardium — a configuration known as the “Spinnaker-sail” or “Angel wing” sign [1, 2].

Although the clinical course is generally mild and self-limiting, such distinctive features pose diagnostic challenges and increase the odds of misinterpretation or unnecessary interventions [3]. We, herein, draw insights from two cases of SPM in full-term neonates exhibiting the “spinnaker-sail” sign. Through an in-depth literature review, we attempt to provide a coherent illustration of the pathophysiology, radiological features, differential diagnosis, and management strategies for clinicians encountering similar cases.

## Case presentation

### Case-1

A 2-day-old boy was brought to our hospital with symptoms of dyspnea. He was born at 38 weeks of gestation via uncomplicated cesarean section. The birth weight was 2550 g. Apgar scores were unavailable. Maternal history included a respiratory tract infection during the second trimester. Antenatal ultrasound findings were insignificant, but routine screening was not conducted.

**Fig. 1:**
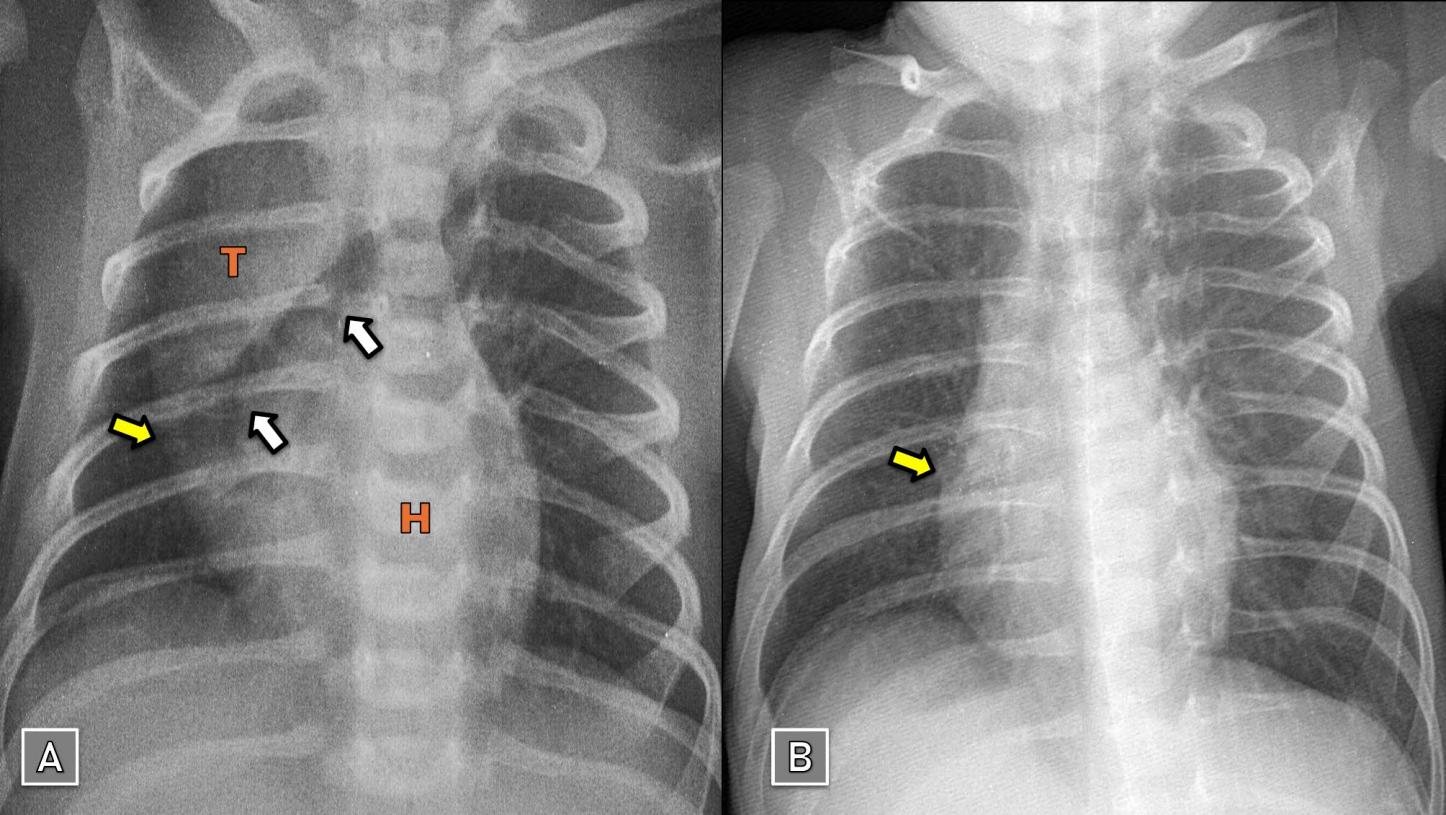
SPM in patient-1: (**A**) Day 2: Spinnaker-sail sign observed. (**B**) Day 11: Near complete resolution. Yellow arrows: lateral borders of the fascia extending over the thymus and pericardium, which trap leaked air and cause outward bulging of the pericardium. White arrows: entrapped air behind this fascia. Blue arrows: leaked air surrounding the mediastinal structures. T: Thymus. H: Heart

**Fig. 2:**
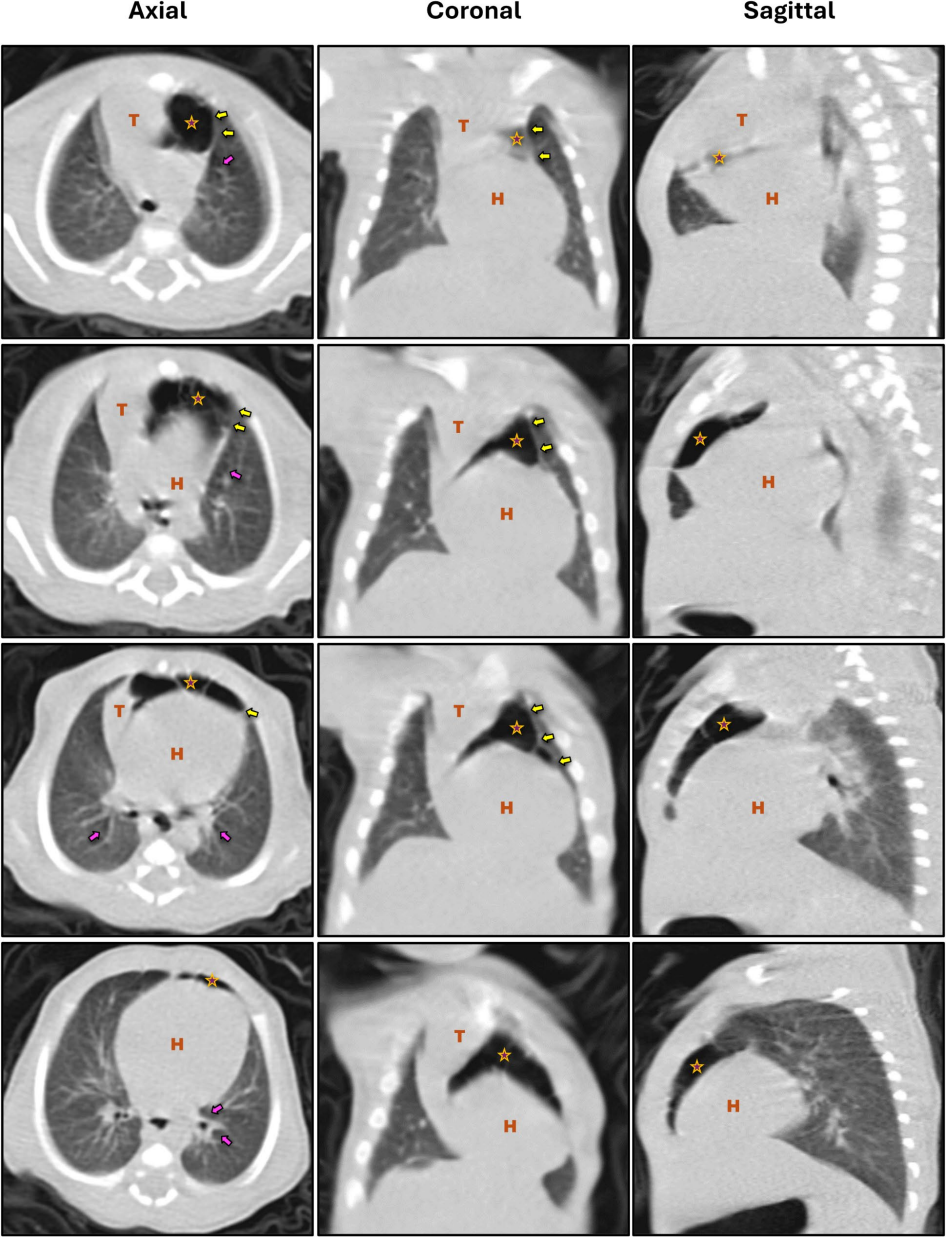
CT of a full-term neonate with SPM (Patient-1). Yellow arrows: the lateral borders of the fascia overlying the thymus and pericardium. Pink arrows: hyperaeration around the main bronchi, indicating air dissection through the peribronchovascular sheaths. The Asterisk (*) multiloculated hyperlucency in the anterior mediastinum displacing the thymus superolaterally, with thin radiopaque lines showing the hyperinflated thymic capsule. (T) Thymus; (H) Heart. All segmental bronchi are within lungs, and no lobar emphysema is noted

Clinical examination revealed tachypnea, nasal flaring, and recessions. Oxygen saturation was 95%. Frontal plane CXR demonstrated a crescent-shaped radiolucent structure separating the thymus from the pericardium, extending from the right lower lobe to the left upper lobe. There were no signs of concomitant pneumothorax or subcutaneous emphysema (Fig. 1A). We performed a CT scan since we lack reliable antenatal US data, it revealed an extrapulmonary multiseptated cystic mass within the anterior mediastinum, in addition to a limited aeration around the bronchi (Fig. 2).

Laboratory tests, including: arterial blood gases, complete blood count, and procalcitonin, were normal. We diagnosed the patient with SPM and admitted him to the NICU. We used oxygen supplementation (1 L/min) and IV-antibiotics. Serial CXR showed a gradual improvement, with near-resolution of the SPM by day 11 (Fig. 1B).

### Case-2

A 6-hour-old boy was admitted to our NICU due to respiratory distress, shortly after birth. He was born at 38 weeks of gestation via uncomplicated cesarean section. The birth weight was 3350 g. Apgar scores were normal. Pregnancy was insignificant, and routine antenatal ultrasound checks were unremarkable.

**Fig. 3:**
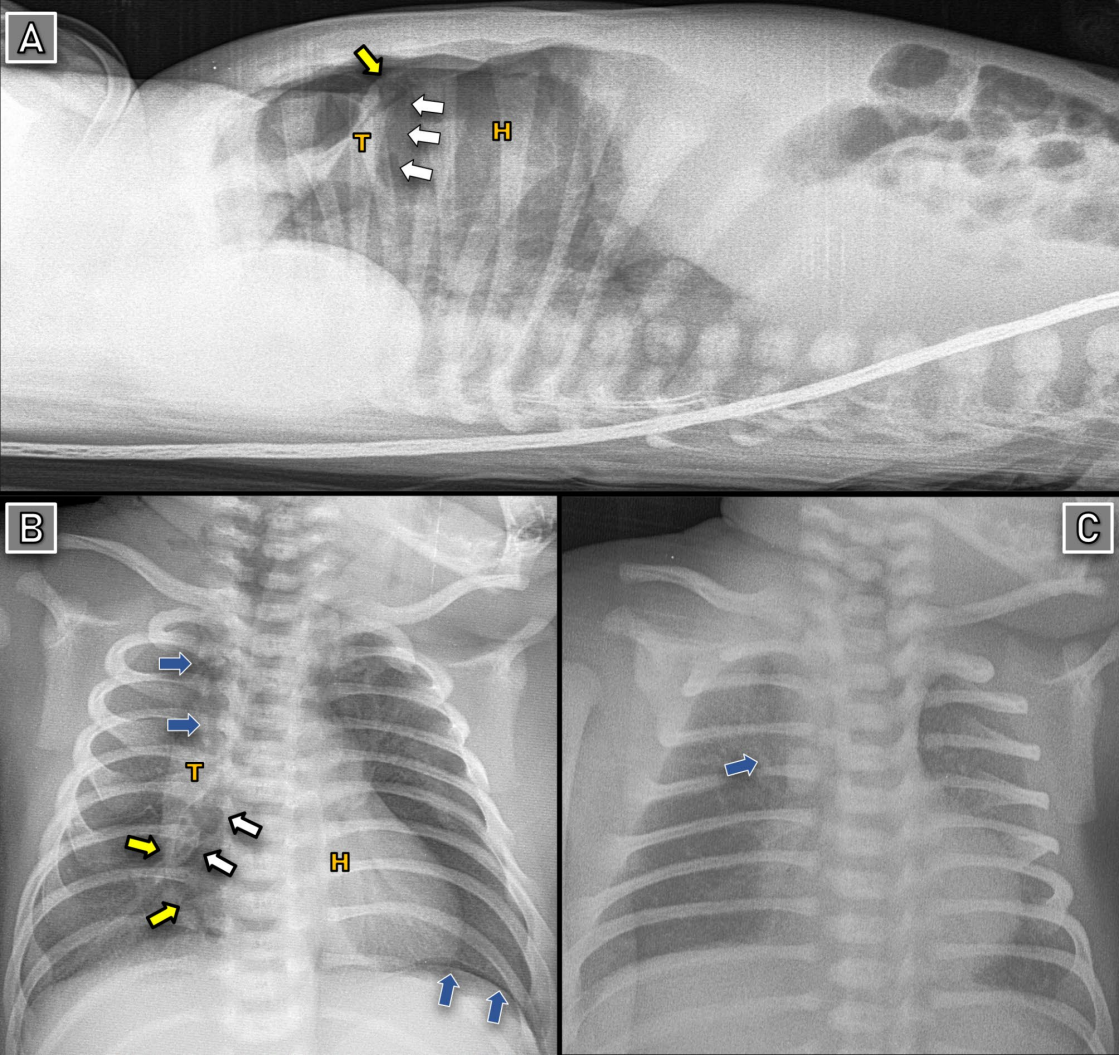
SPM in patient-2: (**A-B**) Day 1: Spinnaker-sail sign and Extrapleural air sign (above left hemidiaphragm) observed. (**C**) Day 4: Near complete resolution. Yellow arrows: lateral borders of the fascia extending over the thymus and pericardium, which trap leaked air and cause outward bulging of the pericardium. White arrows: entrapped air behind this fascia. Blue arrows: leaked air surrounding the mediastinal structures. T: Thymus. H: Heart

24 h later, his respiratory condition deteriorated, which required increasing the oxygen support to 5 L/min. CXR demonstrated a wedge-shaped radiolucent entity separating the thymus from the pericardium, with radiolucent air streaks surrounding the trachea and extending into the neck. Additionally, a radiolucent band overlying the left hemidiaphragm was observed (Extrapleural air sign) (Fig. 3B). A lateral projection CXR confirmed an air collection elevating the thymus from the pericardium and great vessels (Fig. 3A).

Based on these findings, we diagnosed the neonate with SPM and followed up the case with serial CXR. He showed a gradual improvement, with near-resolution of the SPM by day 4 (Fig. 3C).

For both neonates, we used a 45° position when performing CXR to help rule out medial pneumothorax and pneumopericardium. CT in Case-1 and the unremarkable antenatal ultrasonography in Case-2 helped rule out congenital cystic lesions, such as congenital pulmonary adenomatoid malformation, pulmonary sequestration, and congenital lobar emphysema (see Table 2).

## Discussion

Pneumomediastinum (PM) is an uncommon finding in neonates with respiratory pathologies or on mechanical ventilation, estimating an incidence of 4–25 cases per 10,000 live births, with a higher tendency to affect premature infants. Documented risk factors include meconium aspiration, pneumonia, positive-pressure mechanical ventilation, and birth trauma; most of which precipitate an increasing pressure gradient between bronchoalveolar units and the peribronchial or perivascular fasciae [3, 4].

Spontaneous Pneumomediastinum (SPM) in full-term neonates is a rare condition that was first described by Hamman in 1939 [5, 6]. The mechanism is believed to stem from minute ruptures within bronchoalveolar walls, leading to air leakage through the peribronchial and perivascular fasciae into the mediastinum. In the rest of this paper, we will focus only on SPM, drawing insights from two full-term neonates exhibiting the spinnaker-sail sign.

## Anatomy and pathophysiology

Understanding the pathophysiology and radiographic features of SPM lies in recognizing two key-concepts: (A) the sources and mechanisms of air leakage; and (B) the unique anatomy of mediastinal fasciae in neonates.

SPM is believed to stem from a structural immaturity of the alveoli. During labor, vigorous straining against a closed glottis suddenly increases the pressure within these immature bronchoalveolar units, eliciting small tears in their walls as described by Macklin [7] and Hamman [8]. This free air has the capability of breaking into the pleural cavity (pneumothorax), mediastinum (pneumomediastinum), pericardial sac (pneumopericardium), or remains entrapped within perivascular fasciae – cuffing off the blood vessels (pulmonary interstitial emphysema) [2]. Collectively, these sequelae share potential risks of compromising venous return, leading to vascular collapse or sudden death (air block).

Mediastinal viscera and great vessels are naturally invested by distinctive fasciae. Since anatomical textbooks barely discuss the origins and extensions of these layers, we need to elaborate on this concept. The “Pretracheal fascia” was previously viewed as a simple cervical layer extending, anterior to the trachea, into the mediastinum. P Marchand [9] broadened this perception by demonstrating that it is a more complex layer that encompasses both the trachea and esophagus, extends into the mediastinum, and merges with the fibrous pericardium. Laterally, it constitutes a fibrous layer around the bronchi and may extend into the terminal bronchioles, contributing to alveolar septa formation. Thus, ruptured bronchoalveolar walls can leak air through this highway into the mediastinum.

The “Perivascular fascia” is a sheath derived from the fibrous pericardium. It invests the mediastinal great vessels and their extensions into the lungs, constituting an additional pathway for air leakage. These anatomical insights explained the convenient radiographic features of PM, wherein, the leaked air accumulates around the heart, great vessels, trachea, neck, or retroperitoneal spaces [9, 10].

However, the explanation of the “Spinnaker sail sign” (i.e., air entrapped between the thymus and heart) remained unclear until FL Quattromani, et al. [11] published their autopsy findings in neonates both with and without PM. The authors reported a semi-transparent connective tissue that spreads over the anterior surface of the thymus and pericardium, extending from the thoracic inlet to the diaphragm, and laterally merges with the mediastinal pleura. Also, it envelops the thymus and penetrates its lobules as septae. This tissue is believed to be a remnant of a mesenchyme that surrounds the fetal thymus and connects it to the pericardium, facilitating its migration into the anterior mediastinum during fetal development [3, 11]. The authors proposed that this fascia is continuous with the Pretracheal fascia described by P Marchand [9].

The aforementioned findings explain why leaked air is entrapped retrothymically, creating the “spinnaker-sail " sign in neonates, while in older patients, as this fascia and thymus undergo atrophy, PM tends to present differently on imaging [1, 4]. In this paper, we focus on SPM in neonates only. A broader range of etiologies exists for older ages and non-spontaneous forms of PM.

## Clinical and radiographic features

The clinical spectrum of SPM ranges from asymptomatic cases to severe respiratory distress with grunting, cyanosis, hemithorax bulging, or subcutaneous emphysema. As the clinically-silent form is more prevalent and the condition is usually diagnosed incidentally, it is important to discuss the radiographic features of PM [3, 10]. According to the relative laxity of mediastinal fasciae, leaked air can distribute into various mediastinal spaces, resulting in different radiographic patterns [10]. This paper focuses on the Spinnaker-sail sign only, since it is unique to neonates, while Table 1 summarizes the rest of the signs for reference.

**Table 1:** Overview of common radiographic presentations of pneumomediastinum

Sign	Description
Spinnaker Sail	**FP**: Radiolucent crescentic configuration elevates the thymus from pericardium ± thin radiodense line bulging the pericardium outward. **LP**: Air in anterior mediastinum lifts thymus from pericardium and great vessels.
Continuous Diaphragm	**FP**: Thin band of gas between heart and diaphragm * **LP**: Gas outlines the superior surface of hemidiaphragm *
Naclerioʼs V	**FP**: Radiolucent band extends along the descending aorta intersects with band along the medial hemidiaphragm, forming a “V” shape.
Extrapleural Air	**FP**: Radiolucent band extends from the mediastinum and separates pleurae (parietal and visceral layers) from diaphragm.
Ring Around the Artery	**LP**: Lucent ring around extrapericardial segment of pulmonary artery.
Pneumoprecardium	**LP**: Air collection between the heart and sternum.

FP: Frontal Projection. LP: Lateral Projection. (*) Diaphragm is normally obscured by the heart

Chest X-ray (CXR) is a convenient diagnostic tool. The ‘spinnaker-sail sign’ appears on frontal projection as a wedge-shaped radiolucent structure, separating the thymus from the cardiac silhouette. This air outlines the inferior borders of the thymus and displaces it superolaterally [4, 12]. Occasionally, the accumulated air can dissect and inflate the thymic capsule, creating a multiseptated cystic radiolucency. As patients mature, both the thymus and its overlying fascia that entraps leaked air undergo atrophy, which reduces the incidence of this sign in older individuals [4]. The lateral borders of this fascia may be observed as a thin curvilinear radiodense line extending from the inferior pole of the thymus to the heart. Increased pressure of the trapped gas can cause the pericardium where it connects to bulge outward, forming a triangular shape (Figs. 1 and 3) [3, 11]. The lateral CXR projection is often recommended as a reliable next step due to its lower radiation exposure and ease of performance in neonates. Identifying air in the anterior mediastinum elevating the thymus away from the pericardium and great vessels strongly supports the diagnosis of anterior PM [13, 14].

Computed Tomography (CT) provides more comprehensive insights into the architecture of the mediastinal structures, tracheobronchial tree, and lung parenchyma. Identifying all segmental bronchi within the lungs indicates that the air is accumulated extrapulmonary. This point is important to rule out intrapulmonary cystic lesions from the differential diagnosis (discussed below) [3, 4]. Occasionally, air can be observed dissecting through the peribronchial and perivascular sheaths into the mediastinum (Fig. 2). The fascia overlying the thymus and pericardium, described by Quattromani, et al., may also be observed here as a prominent curvilinear line extending from the displaced thymic lobe to the pericardium [4, 11].

Ultrasonography (US) of the mediastinum is a rarely used imaging modality. Air presents as thick echogenic lines surrounding the thymus, within the thymic parenchyma, or between the thymus and the posterior great vessels. However, this technique may miss air collections in certain mediastinal spaces [3, 15].

Chest wall transillumination is also a diagnostic tool with positive outcomes for diagnosing PM, although evidence on its accuracy is limited. This technique involves applying a high-intensity illuminator probe on the chest wall to assess light gradations. Hence, the examiner’s expertise and the neonate’s age, weight, and clarity of the transmitting medium are subjective. It is advocated to spare its use for severe, life-threatening cases that require urgent intervention. Otherwise, it is recommended to obtain a CXR for confirmation [16, 17].

### Differential diagnoses

#### Medial pneumothorax

Medial Pneumothorax (PTX) is a considerable diagnosis when assessing central hyperlucency on CXR [3]. This medial air positioning results from a dorsal falling and rotation of the lung in supine infants, causing intrapleural air to accumulate initially in the anteromedial region. As air builds up, the displacement of the lateral visceral pleural line becomes increasingly evident. In PTX, the mediastinum remains secured by the mediastinal pleura, whereas in PM, air dissects through the mediastinal fasciae, leading to the elevation of the thymus. Tension PTX shifts the mediastinum contralaterally, while tension PM does not. Imaging maneuvers, such as CXR in prone or lateral decubitus positions, can reposition the medial air collection superolaterally — a phenomenon that is not observed in PM. Overall, both conditions may coexist if the mediastinal pleura is ruptured under increasing air tension on either side [10, 18].

#### Pneumopericardium

Pneumopericardium (PPC) poses a diagnostic challenge due to its similar radiographic features, though it is less common in neonates than PM [3, 10]. Symptoms such as sudden-onset bradycardia, muffled heart sounds, and hypotension warrant consideration of PPC. The classic radiographic sign of PPC is a continuous, medially-concave radiolucent band of air outlining the cardiac silhouette, without extending beyond the great vessels. As air accumulates, it develops a single, broad band that encircles the heart and is sharply marginated by the pericardial sac (Halo sign). In contrast, PM typically presents as multiple thin streaks of gas that do not fully encircle the heart; in neonates, it may appear as a crescentic laterally-concave retrothymic radiolucency, i.e. “Spinnaker-sail sign”. Similar to PTX, PPC is position-sensitive. Thus, upright or lateral-decubitus repositioning without relocation of gas suggests PM over PPC [10, 19, 20].

#### Pulmonary interstitial emphysema

Pulmonary Interstitial Emphysema (PIE) is a condition in which the leaked air remains entrapped in the peribronchovascular sheaths and lymphatics, affecting the airways in a local or diffuse manner. It primarily affects underweight, premature, and mechanically ventilated infants. The radiographic presentation progresses over time, beginning with peripheral cystic and linear translucencies on CXR. As the condition advances, a more widespread tubular branching pattern develops, resulting in a hyperexpanded lung with multiple thin-walled cystic and tubular structures that may compromise adjacent tissue or leak air into other cavities [21, 22]. CT imaging shows air extending along the peribronchovascular sheaths and forming characteristic cystic radiolucencies, known as the “line-and-dots” pattern [23]. In contrast, PM occurs when the leaked air dissects through these sheaths into the mediastinal cavities, leading to air accumulation in the mediastinum with far less involvement of the pulmonary interstitium.

Congenital Intrapulmonary cystic lesions are a potential differential diagnosis for neonatal PM. They share some key-features, such as: (1) antenatal US is the main diagnostic tool; (2) clinical presentations can vary from asymptomatic to immediate at birth; and (3) CXR may appear normal despite clinical symptoms.

#### Congenital pulmonary airway malformation & pulmonary sequestration

Congenital Pulmonary Airway Malformation (CPAM) ensues during the pseudoglandular and saccular stages of lung development. It is characterized by excessive growth of the terminal bronchioles and impaired alveolar development, which causes the affected lobe to be enlarged [24]. Stocker, et al. [25, 26] classified CPAM into five subtypes based on the number, size, and epithelial lining of cysts; however, a detailed discussion of these subtypes is beyond the scope of this paper. Postnatal CXR may reveal loculated translucencies and possible mediastinal shift, while CT scans typically show a heterogeneous multilocular cystic mass with thin walls connected to the tracheobronchial tree and supplied by the pulmonary artery. In contrast, Pulmonary Sequestration appears as an intra- or extra-pulmonary homogeneous condensed opacity supplied by aberrant systemic arteries, usually from the descending aorta [24, 27].

#### Congenital Lobar emphysema

Congenital lobar emphysema (CLE) is a rare condition characterized by overinflation of one or more lung lobes due to a one-way valve mechanism. While half of cases are idiopathic, bronchial anomalies and/or obstructions are potentially implicated. Symptoms typically appear within the first few months as respiratory distress and poor feeding [28, 29]. Key findings include hyperresonance on percussion and diminished lung sounds, which can mimic pneumothorax — given the similar radiographic hyperlucency [24]. CXR usually shows a large emphysematous lobe with ipsilateral rib space widening, diaphragm flattening, and adjacent lobe atelectasis. As the condition progresses, herniation and mediastinal shift may occur, potentially causing contralateral lobe atelectasis. This is important as CLE often affects the upper lobes, and herniation can create misleading mediastinal radiolucency. CT is the gold standard for diagnosis, offering detailed exploration of the bronchoalveolar anatomy, lobar hyperaeration or atelectasis, mediastinal shift, and vascular abnormalities [28–31].

Overall, certain variants of cystic neonatal conditions, such as pleuropulmonary blastomas, hybrid lesions (CPAM and BPS), and bronchogenic cysts, may clinically or radiographically mimic Neonatal SPM. Although far less common and not discussed in detail here, these conditions should be considered in differential diagnoses [3, 28]. Table 2 summarizes the key-features of the aforementioned differential diagnosis.

**Table 2:** Radiographic features of neonatal PM and its differential diagnoses

Condition	Radiographic Features	Appendix
PM *	Multiple thin lucent streaks outlining mediastinal structures (trachea, bronchi, great vessels, neck, subcutaneous tissues).	Position does not affect distribution.
PTX	Upright: apical lucency, pleural line. Supine: anteromedial or deep-sulcus lucency; pleural line evident with air accumulation	Position affects air distribution.
PPC	Continuous medially-concave radiolucent band around cardiac silhouette (Halo sign); air-fluid level if effusion present.	Position affects air distribution.
CPAM	Multilocular heterogenous thin-walled intrapulmonary cystic mass, connected to tracheobronchial tree, supplied by the pulmonary artery.	Varies according to subtype.
PS	Homogeneous condensed opacity supplied by aberrant systemic arteries, usually from the descending aorta.	Intrapulmonary or Extrapulmonary
PIE	Peripheral cystic and linear translucencies ◊ widespread tubular branching pattern ◊ multiple thin-walled cystic & tubular structures expanding affected lobe. CT: air along the peribronchovascular sheaths, oriented in a linear or cystic pattern (line-and-dots)	Temporal progression. Localized or diffuse
CLE	Large emphysematous lobe with vascular markings; rib space widening, diaphragm flattening ± adjacent lobe atelectasis or mediastinal shift.	Contralateral lobe atelectasis in advanced cases.

PM: Pneumomediastinum. PTX: Pneumothorax. PPC: Pneumopericardium. CPAM: Congenital Pulmonary Adenomatoid Malformation. PS: Pulmonary Sequestration. PIE: Pulmonary Interstitial Emphysema. CLE: Congenital Lobar Emphysema. (*) see Table 1

### Management and Follow-up

SPM generally resolves spontaneously, often requiring minimal intervention. Management primarily involves NICU admission for supplemental oxygen, vitals monitoring, and follow-up CXR. Empirical antibiotics may be warranted if pneumonia or sepsis is suspected [3, 13].

The rationale behind using supplemental oxygen is based on the “Nitrogen Washout” theory. Atmospheric air consists of approximately 78% nitrogen, 21% oxygen, and other trace gases, resulting in an alveolar total pressure of about 760 mmHg. The total pressure of venous blood gases is around 706 mmHg, creating a pressure gradient that drives gas movement from high-pressure areas (alveoli or pleural cavity) to lower-pressure areas (bloodstream), facilitating PTX resolution. Administering higher oxygen concentrations (e.g., 90–100% FiO2) gradually “washes out” alveolar nitrogen and replaces it with oxygen. As the partial pressure of alveolar nitrogen decreases, the pressure gradient with trapped air in the mediastinal pockets increases, hastening PM resolution. Numerous trials evaluated the efficacy of this maneuver, showing therapeutic benefits in adults, but similar evidence in neonates is lacking. The side effects of hyperoxia — such as V/Q mismatch and absorption atelectasis — complicate incorporating this maneuver in definitive guidelines, particularly for normoxic neonates [32]. Nonetheless, many case reports on neonatal SPM have documented the use of supplemental oxygen in their management [3].

Although rare, tension PM may necessitate a surgical intervention. Needle or catheter decompression is advocated at the second or third intercostal space, just lateral to the sternum, on the side with higher air accumulation. It is noteworthy to be aware of the internal mammary artery course during intervention [33, 34]. In refractory cases, chest tube decompression may be considered, although evidence on this procedure in neonates is limited and inconsistent. JT Moore, et al. [35] suggest a subxiphoid incision for chest tube placement, positioning its tip at the angle of Louis under direct visualization. In older children, a suprasternal notch incision is more commonly reported, with some studies reporting good outcomes when both sites are used simultaneously [36, 37]. Careful blunt dissection is essential to avoid injury to mediastinal structures and to disrupt loculations [35].

Mediastinoscopy has proven to be effective in evaluating the anterior and superior mediastinum in pediatric patients, particularly in the context of malignancies and biopsies, with fewer complications reported in children than adults. However, there is an extreme lack of evidence on its safety and application in neonates [38–40]. Hence, two important questions should be addressed in future research: Is the blunt dissection technique efficient to reach the retrothymic pocket secured by the fetal-remnant fascia that overlies the thymus and pericardium? How safe is to use mediastinoscopy in neonates for this purpose? While our review lays the basis for these questions, it cannot provide the answers.

Low et al., reported a thoracotomy on a neonate with deteriorating distress. The microscopic examination revealed a multiloculated air-filled cystic mediastinal lesion lined with fibrofatty tissue, lacking an epithelial lining [4].

## Conclusions

SPM in full-term neonates poses unique diagnostic challenges, particularly when it presents with rare radiographic patterns, e.g., spinnaker-sail sign. A proper understanding of the distinctive anatomical features of mediastinum in neonates, alongside the mechanisms of air leakage, are important for accurate diagnosis and effective management.

This multiloculated appearance necessitates a careful differential diagnosis, including medial PTX, PPC, CPAM, PS, PIE, CLE, etc. Antenatal US is crucial for identifying congenital cystic anomalies, while postnatal evaluation relies on a combination of imaging modalities, including CXR in multiple positions, CT, or MRI. Conservative management is the cornerstone, while surgical decompression may be indicated in case of deteriorating tension PM. Mediastinoscopy has been shown to be effective in evaluating mediastinal spaces in adults and older children; however, future research should focus on exploring its safety and efficacy in neonates.

## Data Availability

Data sharing is not applicable to this article as no datasets were generated or analyzed during the current study.

## Abbreviations

PM: Pneumomediastinum
SPM: Spontaneous Pneumomediastinum
CXR: Chest X-Ray
CT: Computed Tomography
US: Ultrasonography
PTX: Pneumothorax
PPP: Pneumopericardium
PIE: Pulmonary Interstitial Emphysema
CPAM: Congenital Pulmonary Airway Malformation
PS: Pulmonary Sequestration
CLE: Congenital Lobar Emphysema
